# Severe Myocarditis in the Pediatric Patient: A Simulation for Emergency Department Management

**DOI:** 10.7759/cureus.96862

**Published:** 2025-11-14

**Authors:** Prayerna Uppal, Anita Thomas, Robyn Wing, Lauren Kinneman, Cassandra Koid Jia Shin, Daisy Ciener, Jean I Pearce, Claire Smith, Jennifer Reid, Kimberly Stone, Katina Summerford, Katherine Wolpert

**Affiliations:** 1 Department of Pediatrics, Seattle Children's Hospital, Seattle, USA; 2 Department of Pediatrics, University of Washington School of Medicine, Seattle, USA; 3 Department of Pediatrics, Division of Emergency Medicine, Children's Hospital of Philadelphia, Philadelphia, USA; 4 Department of Emergency Medicine and Pediatrics, Division of Pediatric Emergency Medicine, Brown University, Providence, USA; 5 Department of Pediatrics, Division of Emergency Medicine, Seattle Children’s Hospital, Seattle, USA; 6 Department of Pediatrics, Division of Emergency Medicine, University of Washington School of Medicine, Seattle, USA; 7 Department of Pediatrics, Division of Emergency Medicine, Tulane University School of Medicine, New Orleans, USA; 8 Department of Pediatrics, Division of Emergency Medicine, Louisiana State University Health Sciences Center, New Orleans, USA; 9 Department of Pediatrics, Division of Emergency Medicine, Vanderbilt University Medical Center, Nashville, USA; 10 Department of Pediatrics, Division of Emergency Medicine, Medical College of Wisconsin, Milwaukee, USA; 11 Center for Clinical and Translational Research, Seattle Children's Research Institute, Seattle, USA

**Keywords:** cardiogenic shock, cardiology, emergency medicine, myocarditis, pediatrics

## Abstract

Myocarditis in a pediatric patient can present with a variety of signs and symptoms, leading to a broad differential diagnosis. Delays in the correct identification of myocarditis and the initiation of appropriate interventions can have a detrimental impact on patient outcomes. In this case, a simulated five-year-old patient with myocarditis and secondary cardiogenic shock presents to the emergency department (ED). The goals of this simulation curriculum were to familiarize trainees and ED providers with the often nonspecific presentation of pediatric myocarditis, educate the care team about crucial interventions that positively or negatively impact the patient’s stabilization course, and reinforce the consequences of delays in appropriate interventions, such as progression of cardiogenic shock and potentially fatal arrhythmias.

## Introduction

Severe pediatric myocarditis, or inflammation of the heart muscle, is often accompanied by cardiac dysfunction and is a rare but serious disease presenting to emergency departments. Delayed intervention can cause irreversible cardiovascular injury and death, often the result of disease progression to cardiogenic shock or arrhythmia. Causes of myocarditis include infectious (with a history of a viral prodrome preceding about two-thirds of cases), autoimmune, hypersensitivity reactions, medications, and toxin-mediated mechanisms [1]. While many patients can experience spontaneous recovery, the often vague presentation and extremely high risk of rapid deterioration in severe myocarditis merit provider training and education to improve confidence, efficiency, and the ability to recognize and intervene rapidly while optimizing patient safety. 

This curriculum aims to improve healthcare providers’ ability to correctly identify the diagnosis of severe myocarditis in pediatric patients, minimize time between presentation and appropriate intervention, and increase provider awareness surrounding treatment and complications, including cardiogenic shock and ventricular tachycardia. 

Specifically, this simulation serves as an opportunity for emergency department (ED) teams to recognize interventions that may both worsen the patient’s condition (e.g., intravenous (IV) fluids) in the setting of cardiogenic shock, and reinforce ED providers’ ability to administer high-quality cardiopulmonary resuscitation (CPR). Providers will additionally practice basic principles of Pediatric Advanced Life Support (PALS) following recognition of arrhythmias presented in the scenario.

## Technical report

This simulation occurs in an ED setting with a pediatric patient simulation manikin and requires the use of point-of-care ultrasound (POCUS). Available resources included standard resuscitation equipment as well as imaging modalities, including, but not limited to, POCUS and X-ray. Consultants were available via phone call. Simulation team members included experienced facilitators (usually pediatric emergency medicine (PEM) attendings), a simulation technician, and participants in designated roles. Pending the number of participants and translation to actual clinical roles, the team consisted of a team leader, head of bed/airway provider, bedside nurse, pharmacist, history taker/family communicator, and observers.

The simulation curriculum was developed and run across five academic pediatric emergency departments (PEDs) with seven discrete events and 113 total participants. Events had a minimum of six participants and a maximum of 26 participants. Participants included attending physicians, emergency medicine (EM) residents, PEM fellows, ED nurses, advanced practice practitioners (nurse practitioners and physician assistants), medical students, and pharmacy students. Each institution received environment preparation instructions, the simulation case, a debriefing guide, and a post-simulation survey for each participant to complete. The simulation case included proposed objectives, vital signs, physical exam findings, responses to potential interventions, lab values, and imaging results.

In this simulation, participants complete learning objectives related to resuscitation, medical management, and team management. Participants perform resuscitation skills, including conducting a primary and secondary survey, identifying abnormal cardiac rhythms, performing high-quality CPR, providing appropriate defibrillation, and considering activation of extracorporeal cardiopulmonary resuscitation (eCPR). Medical management learning objectives include identifying cardiogenic shock, managing myocarditis, recognizing ventricular tachycardia, and utilizing PALS algorithms. Participants also complete team management objectives by assigning clear roles and responsibilities, maintaining situational awareness, and using direct, closed-loop communication.

Pre-briefing

Before beginning the simulation, the facilitator conducts any necessary introductions, outlines delegated roles, clearly states the expectations of the session and the importance of a safe learning environment, and orients participants to the available supplies (Table 1).

**Table 1:** Environmental preparation. PALS, Pediatric Advanced Life Support; BP, blood pressure; RR, respiratory rate; SpO₂, oxygen saturation; ETCO₂, end-tidal carbon dioxide; O₂, oxygen; IV, intravenous; IO, intraosseous; CPAP, continuous positive airway pressure

Resources
PALS reference cards, material
Patient Weight Estimator (e.g., Broselow tape)
Pediatric resuscitation medication references (e.g., Broselow tape)
Code documentation forms
Universal precautions
Gloves
Gowns
Masks
Medications (consider having all or only a limited number of medications available)
Antibiotics:
Ceftriaxone
Vancomycin
Antipyretics:
Acetaminophen
Ibuprofen
Fluids:
Normal saline (warmed or room temperature)
Hypertonic saline (3%)
Lactated Ringer's (warmed or room temperature)
Vasopressors
Epinephrine
Norepinephrine
Dopamine
Milrinone
Dobutamine
Resuscitation and intubation:
Atropine
Epinephrine
Etomidate
Fentanyl
Ketamine
Propofol
Rocuronium
Succinylcholine
Equipment
Simulator on bed with patient identification band
Monitor - BP, HR, RR, SpO2, temperature and ETCO2 monitor (if available)
Blood pressure cuff
Heart rate monitor leads.
Oxygen saturation probe
ETCO2 cannula (if available)
Oxygen hook-up on the wall or a cylinder
Bag-mask system, multiple-size masks
O2 - nasal cannula, mask - simple and/or non-rebreather
Suction
Thermometer, temperature probe
Nasal, oral airways, multiple sizes
Endotracheal tubes - 3.0, 3.5, 4.0, 4.5, 5.0, cuffed or uncuffed, stylets
Laryngoscope, Miller and Mac blades, multiple sizes
End-tidal CO2 colorimeter
Nasogastric tube(s)
Stethoscopes
IV/Angiocatheter, various sizes
IO needles, 2 sizes
Gauze, Tape
IV tubing/blood product tubing and filters
IV pumps, pressure bags/ blood product pumps
Syringes, multiple sizes
Bedside blood sample processors: glucose, electrolytes, gases
Specimen tubes
Crash cart and backboard
Defibrillator
High flow nasal cannula system and/or nasal CPAP system

Case

**Figure 1:**
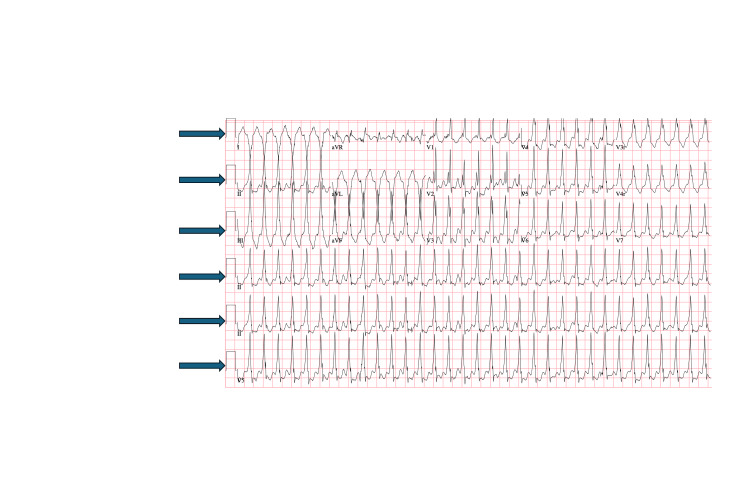
Electrocardiogram (EKG). Arrows demonstrating ventricular tachycardia in the EKG of a pediatric patient. Courtesy of the authors.

**Figure 2:**
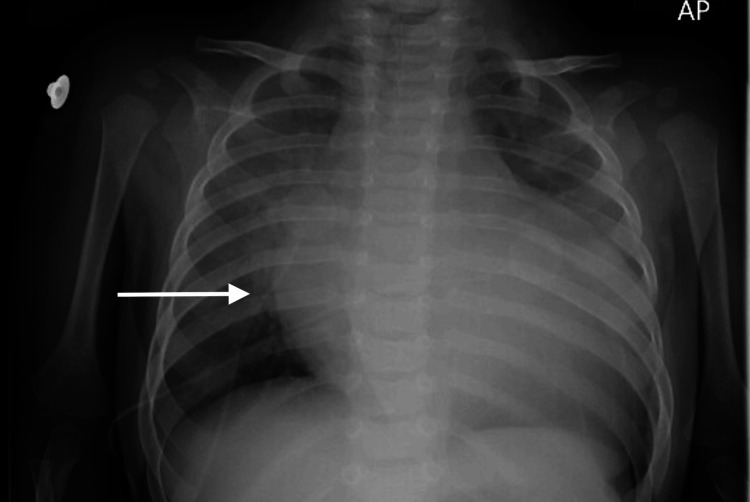
Chest X-ray. Arrow indicating cardiomegaly on the chest X-ray of a patient presenting with signs and symptoms concerning for myocarditis and cardiogenic shock. Courtesy of the authors

The simulated case began with an ill-appearing five-year-old male patient brought to the ED by his parents for a two-day history of fever, cough, vomiting, and diarrhea. He had poor oral intake, low urine output, and new-onset tachypnea. The facilitator introduced the scenario outside the simulation area (Table 2). The participants then proceeded to the room for their initial evaluation, and the facilitator revealed the patient’s medical history, physical examination findings, and vital signs as prompted by the participants (Tables 2-3). After obtaining an initial survey and set of vital signs, the simulation progressed based on participant interventions (Table 4). Laboratory results (Table 5), cardiac POCUS images (Videos 1-5), an electrocardiogram (Figure 1), a chest X-ray (Figure 2), and consultations were made available by the facilitator as requested.

**Table 2:** Facilitator guide to the initial presentation. ED, emergency department; HR, heart rate; bpm, beats per minute; SpO₂, oxygen saturation; BP, blood pressure; RR, respiratory rate; T, temperature

Item	Description	Additional information
History of present illness	You are working in the ED when the lobby nurse tells you she just put an ill-appearing five-year-old boy in the resuscitation room. The patient was brought in by his parents with a two-day history of fever, cough, vomiting, and diarrhea. He has not been eating well and has had no urine output for the last 12 hours. Today, his breathing has been fast, which prompted the parents to bring him to the ED. The lobby nurse was concerned and brought him right back.	The team is given the introduction outside of the room. The patient is initially clothed, with no triage vitals, and is not on monitors.
Past medical history	Full term. No medications daily. No known documented allergies. Up to date on immunizations.	The facilitator provides this information if prompted.
Vital signs	HR 184 bpm, Sp02 92%, BP 90/36, RR 42, T 38.6 C	Vital sign values are provided as the team places the patient on respective monitors.

**Table 3:** Facilitator guide to the initial presentation - physical parameters. HEENT, head, ears, eyes, nose, throat

Physical exam	
General	Lethargic, lying in bed, weak cry
HEENT	Eyes open
Respiratory	Tachypneic with grunting noises. Diffuse crackles and rhonchi
Cardiovascular	Tachycardic, a gallop is appreciated. Mottling with a capillary refill time of three seconds
Abdominal	The liver edge is not palpable
Skin	No signs of trauma
Neurologic	Poor tone, minimal withdrawal to pain

**Table 4:** Case progression. ED, emergency department; HR, heart rate; bpm, beats per minute; SpO₂, oxygen saturation; BP, blood pressure; RR, respiratory rate; T, temperature; IV, intravenous; IO, intraosseous; CXR, chest X-ray; POCUS, point-of-care ultrasound; CPR, cardiopulmonary resuscitation; PALS, Pediatric Advanced Life Support; EKG, electrocardiogram; PICU, pediatric intensive care unit; ROSC, return of spontaneous circulation; BVM, bag-valve mask; CICU, cardiac intensive care unit; PEA, pulseless electrical activity; V-tach, ventricular tachycardia

Stage/Time point	Change in case	Additional information
Stage #1. The patient is evaluated in the ED room/0-2 minutes	Participants should first perform the primary survey. The patient is lying down, lethargic, has a weak cry, poor tone, and minimal withdrawal to pain. His exam is significant for tachypnea, grunting, diffuse crackles, rhonchi, tachycardia, and a gallop. The liver edge is not palpable, and the patient is mottled with a capillary refill of three seconds. There are no visible signs of trauma. Vital signs demonstrate an HR of 184 bpm, SpO2 92% on room air, BP 90/36 mmHg, RR 42, and T 38.6 °C. After identifying that the patient is in shock, participants should begin management with supplemental oxygen, obtain IV or IO access, and begin judicious fluid resuscitation with 5-10 ml/kg of crystalloid. The first IV attempt is unsuccessful, prompting a successful second IV or first IO placement. I-Stat labs should be obtained, with results available within one minute. Labs include a venous blood gas with a pH of 7.13, a partial pressure of carbon dioxide of 42 mmHg, a bicarbonate of 13 mmol/L, and a base deficit of 6 mmol/L. The patient’s glucose is 52 mg/dL, and lactate is 3 mmol/L.	If a crystalloid bolus is started, move to the next stage. If no bolus is given, move to the next stage after two minutes. If prompted for a CXR or subspecialist consultation (e.g., PICU or Cardiology), the facilitator will respond that “they are on their way.”
Stage #2. The patient experiences worsening cardiogenic shock/2-5 minutes	The patient clinically decompensates. He has a weaker cry, weak movements, poor tone, and minimal withdrawal to pain. In addition to his initial pulmonary and cardiac exam findings, his liver edge is now palpable below the costal margin, and his capillary refill is further delayed to four seconds. Vital signs at this stage include a HR of 196 bpm, SpO2 89% if supplemental oxygen has been placed or 82% if supplemental oxygen has not been started, BP 80/34 mmHg, RR 50, and T 38.6 °C. Participants should recognize worsening cardiogenic shock. Decompensation should prompt participants to consider cardiogenic shock and perform a cardiac POCUS exam. At this point, participants should consider the initiation of inotropes (e.g., milrinone) or vasopressors (e.g., epinephrine, dopamine).	The patient will immediately decompensate after 20 mL/kg of fluid is given. If no bolus or a single 5-10 mL/kg bolus is given, then the patient will slowly decompensate over the course of 1 minute. Cardiac POCUS demonstrates dilated heart chambers and hypokinesis. Labs available, CXR is “on the way.” Consultants are “on their way." After three minutes of management, move to Stage #3.
Stage #3. Patient decompensates into ventricular tachycardia (VTach) arrhythmia/5-14 minutes	Despite any intervention thus far, the patient suddenly becomes apneic and unresponsive without palpable pulses. Capillary refill is now eight seconds, and the monitor demonstrates ventricular tachycardia. Vital signs at this stage include an HR that can be determined with ventricular tachycardia on the monitor, a SpO2 that is unable to be detected, a BP that is unable to be determined, a RR of 0, and a T 38.6 °C. Participants should recognize pulseless ventricular tachycardia and initiate CPR via the PALS algorithm. An EKG is available. Participants should perform airway management, defibrillation, and code activation. After two defibrillations and one dose of epinephrine, the patient remains in a pulseless rhythm of ventricular tachycardia. Following the third defibrillation, his rhythm returns to sinus tachycardia.	EKG is available if it is requested. EKG demonstrates ventricular tachycardia. If allotted simulation time does not allow for three shocks, convert back to sinus tachycardia after 1st or 2nd defibrillation. If asked, “ The PICU is on their way,” and “Cardiology will call back.”
Stage #4. Patient has ROSC/14-16 minutes	After ROSC, the patient improves, and post-arrest care should be initiated. He now withdraws to pain, has weak palpable pulses, and capillary refill is four seconds. Vitals improve with an HR of 176 bpm, SpO2 96% via BVM and 89% if not receiving BVM respiratory support, BP 65/38 mmHg, RR will be BMV rate (if no support, RR 10), and T 38.6 °C. Blood pressure should be supported with consideration of ionotropic (e.g., milrinone) and/or vasopressor support (e.g., epinephrine or dopamine).	If asked, “PICU is on their way,” and “Cardiology will call back.” After two minutes of post-arrest care, move to Stage #5.
Stage #5. Continued cardiogenic shock after resolution of VTach/16-21 minutes	The patient continues to exhibit signs of cardiogenic shock. On exam, he withdraws and localizes to pain, continues to have weak pulses, and has a four-second capillary refill. Vital signs demonstrate an HR of 150 bpm, SpO2 96% via BVM (or RR 10 if no support), BP 75/40 mmHg, and T 38.6 °C. Participants should recognize the high risk of intubation leading to cardiac arrest in this patient. CXR becomes available, and cardiac POCUS may be performed or repeated.	Cardiology calls back and recommends admission to the CICU. CXR becomes available and demonstrates cardiomegaly. Cardiac POCUS demonstrates dilated heart chambers and hypokinesis. If the team attempts intubation, move to Stage # 6. If no attempt at intubation or if the patient was intubated during CPR in Stage # 3, end the scenario with “CICU has a bed ready and the patient will be transferred for further care.”
Stage #6. Cardiac arrest in the setting of high-risk intubation/21-26 minutes	While attempting intubation, the patient becomes bradycardic on the monitor and develops PEA. The patient is apneic, pulseless, limp, and unresponsive with a capillary refill of seven seconds. The stage starts with an HR of 45 bpm on the monitor, SpO2 96% with BVM, RR via BVM, BP 65/30 mmHg, and T 38.6 °C. Participants should identify PEA and initiate CPR. The PALS algorithm for PEA should be followed. After a single round of CPR and a dose of epinephrine, the patient will achieve a return of spontaneous circulation.	After ROSC is achieved, move to Stage #7.
Stage #7. ROSC in patient after PEA/26-28 minutes	The patient has ROSC and, on repeat examination, has palpable pulses. Vital signs demonstrate an HR of 90, SpO2 96% on supplemental oxygen, RR via BVM, BP 85/50 mmHg, and T 38.6 °C. He is in a normal sinus rhythm. The scenario will end as the team verbalizes the need to initiate post-resuscitation care and transfer the patient to the critical care unit.	“CICU bed is ready,” and the patient will be transferred for further care. This marks the end of the case.

**Table 5:** i-STAT labs. CO2, partial pressure of carbon dioxide; HCO3, bicarbonate

Lab	Value	Reference range
pH	7.13	7.33-7.43
CO2	42 mmHg	40-50 mmHg
HCO3	13 mmol/L	22-29 mmol/L
Base deficit	-6	-2 to +2
Glucose	52 mg/dL	70-100 mg/dL
Lactate	3 mmol/L	0.30-2.00 mmol/L

**Video 1:** POCUS demonstrating dilated heart chambers and hypokinesis (apical four-chamber view). Courtesy of the authors. POCUS, point-of-care ultrasound

**Video 2:** POCUS demonstrating dilated heart chambers and hypokinesis (parasternal long-axis view). Courtesy of the authors. POCUS, point-of-care ultrasound

**Video 3:** POCUS demonstrating dilated heart chambers and hypokinesis (parasternal short-axis view). Courtesy of the authors. POCUS, point-of-care ultrasound

**Video 4:** POCUS demonstrating dilated heart chambers and hypokinesis (subcostal four-chamber view). Courtesy of the authors. POCUS, point-of-care ultrasound

**Video 5:** POCUS demonstrating dilated heart chambers and hypokinesis (subcostal inferior vena cava view). Courtesy of the authors. POCUS, point-of-care ultrasound

Debriefing

After the simulation concluded, facilitators led participants through a 30-to 45-minute debriefing session reviewing both successful and challenging aspects of the case, major learning points gleaned from the experience, and ways in which the case could be improved. Debriefs were guided by provided materials (Table 6), and included the following stages: initial reactions, reviewing medical management and technical skills required for the case, discussing teamwork and communication skills, and a summary of the discussion. Medical management and technical skills specific to myocarditis were reviewed, with a focus on primary survey of Airway, Breathing, Circulation, Disability, Exposure (ABCDEs), avoiding premature diagnostic closure, recognizing pediatric myocarditis, and acknowledging that it can be a difficult diagnosis to make, as well as initial management of cardiogenic shock.

**Table 6:** Debriefing guide. IV, intravenous; IO, intraosseous; IVC, inferior vena cava; CPR, cardiopulmonary resuscitation; eCPR, extracorporeal cardiopulmonary resuscitation

Recognizing myocarditis	
Debriefer script	Discussion points
I noticed you (were quick/took a while) to include myocarditis on your differential. Tell me more about this. What were your thought processes around what was occurring? What helped/hindered you in creating an assessment and plan?	Identify the risks/signs of myocarditis.
	Identify preceding symptoms
	Consider vital signs and subsequent decompensation after initial fluid bolus.
Initial identification and management of myocarditis	
I noticed you (were quick/ could have been quicker) to recognize worsening shock after the initial fluid bolus. This was (great/could lead to delays) in clinical stabilization. How did your team decide on the management priorities? OR I noticed you (were complete/missed some opportunities) in your initial evaluation of this patient in shock. This was (great/could have been even better) because early identification and management could lead to improved outcomes. How did your team decide on the evaluation priorities, and how did your team determine what kind of shock this patient was in? What helped/hindered you?	Initial management of cardiogenic shock, including IV/IO access
	Consideration for a judicious fluid bolus
	Utility of cardiac POCUS to evaluate contractility, IVC
	Consider X-ray imaging for cardiomegaly.
	Consider inotropic support (e.g., Milrinone), especially in post-cardiac surgery patients and in cases with impaired right ventricular function and/or associated pulmonary arterial hypertension.
	Consider vasopressor support. Epinephrine is used in patients with low cardiac output, reduced vascular resistance, and persistent hypotension.
I noticed you (were quick/ could have been quicker) to recognize the progression to pulseless ventricular tachycardia. This was (great/could lead to delays) in clinical stabilization. What helped/hindered you? OR I noticed you (were complete/missed some opportunities) in your initial evaluation of pulseless ventricular tachycardia. This was (great/could have been even better) because early identification and management could lead to improved outcomes. How did your team decide on the evaluation priorities, and what led to the determination of the rhythm change?	Initial management of pulseless ventricular tachycardia
	Components of high-quality CPR
	Importance of defibrillation with 2 and 4 J/kg
	Epinephrine administration
	Consideration of an advanced airway
	Consider activation of eCPR.
I noticed you (were complete/missed some opportunities) in your post-arrest care. This was (great/could have been even better) because effective post-arrest management can lead to better outcomes. How did your team decide on the evaluation priorities post-arrest? What made you (pursue or not pursue) intubation?	Initiate Post-Arrest Management
	Reassess airway, breathing, circulation. Ensure adequate ventilation and maintain appropriate blood pressures.
	Determine and facilitate disposition.
	Recognize that this patient is a high risk of intubation.

Post-simulation survey

Following the debrief, participants were asked to complete a post-simulation survey (Appendix). Utilizing a Likert scale, participants were asked whether they agreed with each statement (1 = strongly disagree, 2 = disagree, 3 = neutral, 4 = agree, 5 = strongly agree). These questions prompted participants to reflect on the simulation processes and the clinical decision-making involved in the evaluation of a pediatric patient with myocarditis, as described in the learning objectives. The survey also included open-ended questions to allow participants the opportunity to identify how this simulation will impact their care and a space for participants to provide feedback on the simulation scenario for future improvement.

Results

Of the 113 participants who completed the scenario, 112 participants completed all questions on the post-simulation survey. The excluded participant did not participate in the debrief and was unable to complete all questions. Participants included eight attending PEM physicians, 16 PEM fellow physicians, 56 EM resident physicians, five nurses, seven medical students, two advanced practice providers, and one pharmacy student. Seventeen participants did not provide demographic data on training year and/or discipline. Cumulative numerical survey responses are presented in Table 7. Summative themes of participant comments to open-ended questions are included in Tables 8-9.

**Table 7:** Cumulative numerical participant responses to post-simulation survey questions. *Rated on a five-point Likert scale: 1 = strongly disagree, 2 = disagree, 3 = neutral, 4 = agree, 5 = strongly agree.

Statement	Strongly agree, *n* (%)	Agree, *n* (%)	Neutral, *n* (%)	Disagree, *n* (%)	Strongly disagree, *n* (%)
This simulation case provided is relevant to my work.	101 (90%)	11 (10%)	0 (0%)	0 (0%)	0 (0%)
The simulation case was realistic.	89 (79%)	22 (20%)	1 (1%)	0 (0%)	0 (0%)
This simulation case was effective in teaching basic resuscitation skills.	97 (87%)	15 (13%)	0 (0%)	0 (0%)	0 (0%)
I feel prepared to perform a primary survey on a patient with cardiogenic shock.	66 (59%)	35 (39%)	10 (9%)	1 (1%)	0 (0%)
This scenario prepared me to elicit critical history from a patient with myocarditis.	61 (54%)	39 (35%)	11 (10%)	1 (1%)	0 (0%)
I feel comfortable activating team assistance early in a resuscitative event.	70 (63%)	35 (31%)	7 (6%)	0 (0%)	0 (0%)
This scenario allowed me to practice effective teamwork and communication skills.	84 (75%)	22 (20%)	6 (5%)	0 (0%)	0 (0%)
I can recognize the presentation of myocarditis.	51 (46%)	48 (43%)	12 (11%)	1 (1%)	0 (0%)
This simulation case was effective in teaching myocarditis management skills.	84 (75%)	25 (22%)	3 (3%)	0 (0%)	0 (0%)
I feel equipped to discuss the management of myocarditis families.	57 (51%)	33 (29%)	19 (17%)	3 (3%)	0 (0%)
The debrief created a safe environment.	105 (94%)	7 (6)	0 (0%)	0 (0%)	0 (0%)
The debrief promoted reflection and team discussion.	104 (93%)	8 (7%)	0 (0%)	0 (0%)	0 (0%)

**Table 8:** Themed participant responses to the post-simulation survey question “How will this simulation change how you do your job?”

Theme	Representative quotes
The importance of keeping myocarditis on the differential diagnosis	“Always have myocarditis on differential when tachycardic and dyspneic.”
	“Keeping myocarditis on differential”
	“Considering myocarditis”
	“Will help me recognize myocarditis or at least have it on the differential.”
The importance of early point-of-care ultrasound (POCUS)	“US quick”
	“Think about POCUS early.”
	“Using POCUS in peds more”
	“More likely to POCUS heart”
The importance of reassessment after interventions	“Ensure to monitor patient exam after fluid resuscitation to evaluate for other less common causes of shock other than septic or hypovolemic shock.”
	“Pivoting early when interventions aren’t working as expected”
	“Reassess after every intervention.”
Management of cardiogenic shock	“Management of pediatric shock, particularly cardiogenic”
	“Improve resuscitation of pediatric cardiogenic shock”
	“Dobutamine and inotropic support early, cardiogenic shock”
	“Choosing proper pressor support for cardiogenic shock”
	“Inotropes early in peds cardiogenic shock”
Importance of recognizing abnormal vital signs in the evaluation of a pediatric patient	“Always have myocarditis on differential when tachycardic and dyspneic.”
	“Recognize myocarditis as a cause of tachycardia and fever in kids”
	“Pay attention to heart rate in any viral child.”
	“POCUS on peds patients who are tachy and hypotensive”
Importance and use of Pediatric Advanced Life Support (PALS) algorithms in pediatric emergencies	“Pediatric CPR review”
	“Keep ACLS, PALS cards with them.”
	“PALS card out!”
Importance of early activation of extracorporeal cardiopulmonary resuscitation (eCPR/ECMO)	“Call for ECMO early.”
	“Early ECMO activation”
Clear communication in a resuscitation	“Steps for shared mental model and improving care”
	“Reminder to assign role to speak with parent during a code or when there is a critically ill patient.”
	“Reinforces closed loop communication”

**Table 9:** Themed participant responses to the post-simulation survey question “How can this scenario be improved?"

Theme	Representative quotes
Too rapid a decline in patient progression	“We had the ultrasound at the bedside within minutes and it felt like the faster we were suspicious, the faster the patient would decompensate.”
	“Maybe a more progressive decline”
	“Longer time frame for the simulation and team members allowing each other to make more mistakes”
	“Give 5 more minutes for completion of scenario.”
Improving ancillary studies	“More response or labs”
	“EKG would be helpful.”
Improving interdisciplinary team roles	“Designating roles prior to starting or having nursing staff self delegate roles”
	“Family for history”
	“Felt like there was a delay with nursing between assessing for interventions and getting updated clinical picture.”
	“CPR coach”
	“Having nurses act as nurses instead of residents acting as nurses”
No changes	“Great megacode!”
	“Perfect”
	“I thought this was great.”

The majority of participants strongly agreed that the scenario was relevant and realistic to their practice. Furthermore, participants strongly agreed the curriculum was effective in teaching myocarditis management. All participants agreed or strongly agreed that the debrief created a safe environment and promoted reflection and team discussion. 

Regarding the open-ended questions at the end of the survey, nine participants highlighted that POCUS was an important adjunct in their learning and assessment of this patient case. Two participants recommended that additional supportive materials, including more labs and EKGs would be helpful in future iterations. Additionally, three participants commented that the rate of patient decline could be more gradual, while two participants felt the scenario required more time than was allotted. 

## Discussion

This simulated scenario aimed to provide participants with an opportunity to recognize and manage cardiogenic shock in a pediatric patient presenting with myocarditis. Originally intended for PEM and EM trainees, participants in the curriculum included attending physicians, ED nurses, and advanced practice providers, medical students, and pharmacy students, demonstrating that a larger interdisciplinary audience benefited from learning in the safe space of this simulated scenario. Across all disciplines, the participants reported that the scenario was realistic, relevant to their work, and effectively taught resuscitation skills. After the scenario, the majority of participants felt they were able to recognize and manage myocarditis in a pediatric patient. Point-of-care ultrasound, an important tool incorporated into this curriculum, was independently highlighted by many in the open-ended questions of the post-simulation survey as an incredibly useful resource in the evaluation of the decompensating pediatric patient. Provided with dynamic ultrasound images during the scenario, participants received exposure to the sequelae of myocarditis, leading to poor cardiac function. In the decompensating pediatric patient where congenital heart disease is not suspected, POCUS can be utilized in conjunction with patient presentation to guide therapeutic interventions [2]. In the literature, cardiac POCUS has been acknowledged as an effective tool in the evaluation of pediatric hemodynamics and shock [2-5]. Cardiac POCUS can also assess left ventricular function [2,3,6,7] and be utilized for evaluation of pericardial effusion [2,7].

Feedback from participants was implemented to improve the case. Labs, an EKG, and additional stages to promote a slower decline in patient progression were incorporated based on participant comments. Time management by facilitators will be important to consider going forward, as case progression will ideally allow all objectives to be completed within a facilitator’s allotted time frame. At least one hour is suggested to complete the curriculum and debrief (30 minutes each); however, future iterations could consider extending the time allotted to 90 minutes or 75 minutes (with 45 minutes for the simulation case, followed by a 30-minute debrief). If a shorter scenario is required, a facilitator may consider stopping the case after Stage #4 (Table 4). This would maintain the case's high educational value by allowing participants to resuscitate and medically manage a patient with myocarditis in an abbreviated time frame.

With a wide range of training among participants, it can be expected that their ability to manage a complex diagnosis will be variable. A limitation of this study is that participant survey responses do not directly evaluate proficiency and understanding. To better understand participants’ knowledge before the simulation and their ability to manage a patient with myocarditis after the simulation, it would be helpful to administer pre- and post-simulation surveys assessing knowledge. Participant pre- and post-survey expectations could be covered in the pre-brief to maintain a safe learning environment. Additionally, limitations exist when utilizing the Likert scale as the primary assessment tool, including response bias and/or acquiescence bias [8]. Another limitation includes the variability in performing simulations between institutions. Different personnel and equipment may create different experiences within the scenario; however, the outlined guides and materials aim to decrease variation in conducting the case.

## Conclusions

Myocarditis is a rare and potentially life-threatening illness in pediatric patients presenting to EDs. This simulation curriculum provides a safe learning environment for interdisciplinary ED providers to build and practice the skills necessary to promote timely diagnosis, perform effective management, and promote the best outcomes for this patient population. In future iterations of the scenario, a pre- and post-simulation survey may help assess knowledge gained through participation. Carrying this curriculum and research forward to reach a broad audience will be important, as it affords continued education in recognizing and managing a rare but serious diagnosis in pediatric patients presenting to EDs.

## Appendices

Appendix: Post-simulation survey

**Table 10:** Post-Simulation Survey

	Strongly Disagree	Disagree	Neutral	Agree	Strongly Agree
This simulation case provided is relevant to my work.	1	2	3	4	5
The simulation case was realistic.	1	2	3	4	5
This simulation case was effective in teaching basic resuscitation skills.	1	2	3	4	5
I feel prepared to perform a primary survey on a patient with a cardiogenic shock.	1	2	3	4	5
This scenario prepared me to elicit critical history from a patient with myocarditis.	1	2	3	4	5
I feel comfortable activating team assistance early in a resuscitative event.	1	2	3	4	5
This scenario allowed me to practice effective teamwork and communication skills.	1	2	3	4	5
I can recognize the presentation of myocarditis.	1	2	3	4	5
This simulation case was effective in teaching myocarditis and arrhythmia management skills.	1	2	3	4	5
I feel equipped to discuss management of myocarditis families.	1	2	3	4	5
The debrief created a safe environment.	1	2	3	4	5
The debrief promoted reflection and team discussion.	1	2	3	4	5
